# Hydrogel Delivery System Containing *Calendulae flos* Lyophilized Extract with Chitosan as a Supporting Strategy for Wound Healing Applications

**DOI:** 10.3390/pharmaceutics12070634

**Published:** 2020-07-07

**Authors:** Justyna Chanaj-Kaczmarek, Magdalena Paczkowska, Tomasz Osmałek, Barbara Kaproń, Tomasz Plech, Daria Szymanowska, Marta Karaźniewicz-Łada, Joanna Kobus-Cisowska, Judyta Cielecka-Piontek

**Affiliations:** 1 Department of Pharmacognosy, Poznan University of Medical Sciences, 4 Swiecickiego Street, 60781 Poznan, Poland; justyna.chanaj-kaczmarek@ump.edu.pl (J.C.-K.); mpaczkowska@ump.edu.pl (M.P.); 2 Department of Pharmaceutical Technology, Poznan University of Medical Sciences, 6 Grunwaldzka Street, 60780 Poznan, Poland; tosmalek@ump.edu.pl; 3 Department of Clinical Genetics, Medical University of Lublin, 11 Radziwillowska Street, 20080 Lublin, Poland; barbara.kapron@umlub.pl; 4 Department of Pharmacology, Faculty of Health Sciences, Medical University of Lublin, 4a Chodzki Street, 20093 Lublin, Poland; tomaszplech@umlub.pl; 5 Faculty of Food Science and Nutrition, Poznan University of Life Sciences, 31 Wojska Polskiego Street, 60-634 Poznan, Poland; daria.szymanowska@up.poznan.pl; 6 Department of Physical Pharmacy and Pharmacokinetics, Poznan University of Medical Sciences, 6 Swiecickiego Street, 60781 Poznan, Poland; mkaraz@ump.edu.pl; 7 Department of Gastronomy Science and Functional Foods, Poznan University of Life Sciences, Wojska Polskiego 28, 60637 Poznan, Poland; joanna.kobus-cisowska@up.poznan.pl

**Keywords:** *Calendula officinalis* L., chitosan, delivery systems, adhesion, topical

## Abstract

*Calendulae flos* is a valued plant material with known anti-inflammatory and antimicrobiological properties. The limitation for its use in the treatment of chronic wounds is the lack of adhesion to the required site of action. Obtaining the *Calendulae flos* lyophilized extract from water-ethanolic extract allowed to prepare valuable material whose biological activity in the wound healing process was confirmed by a positive result of the scratch test. The *Calendulae flos* lyophilized extract was standardized for the contents of the chlorogenic acid and the narcissin. The significant potency of the *Calendulae flos* pharmacological activity has become the reason for studies on its novel applications in combination with the multifunctional chitosan carrier, to create a new, valuable solution in the treatment of chronic wounds. The use of chitosan as a carrier allowed for the controlled release of the chlorogenic acid and the narcissin. These substances, characterized by prolonged release from the chitosan delivery system, were identified as well permeable, based on the results of the studies of the parallel artificial membrane permeability assay (PAMPA Skin) a model simulating permeability through membrane skin. The combination of the *Calendulae flos* lyophilized extract and the chitosan allowed for synergy of action towards hyaluronidase inhibition and effective microbiological activity. Optimization of the hypromellose hydrogel preparation ensuring the required rheological properties necessary for the release of the chlorogenic acid and the narcissin from the chitosan delivery system, as well as demonstrated antimicrobial activity allows indicating formulations of 3% *Calendulae flos* lyophilized extract with chitosan 80/500 in weight ratio 1:1 and 2% or 3% hypromellose as an important support with high compliance of response and effectiveness for patients suffering from chronic wounds.

## 1. Introduction

Chronic wounds are an important health problem that affects many people around the world. It is estimated that currently, 6.5 million in the USA, and 2.0 million patients in the EU suffer from chronic wounds. The annual wound care products market is projected to reach $15–22 billion by 2024 [1]. The aging of the world population and the increase in the frequency of civilization diseases will influence the increasing percentage of patients suffering from chronic wounds.

Wound healing has multiple pathophysiological processes in which the skin and the tissues under it repair themselves after injury. Wound healing involves three biological stages: (i) inflammation, which usually lasts up to six days, (ii) proliferation, which typically covers the following two weeks, and (iii) remodeling, which continues for up to two years [2]. Extending the healing period of the injury over 4–6 weeks is considered the basis for the diagnosis of a chronic wound [3]. Invasive surgical methods or innovative wound dressings, used in the treatment of chronic wounds, are available only to a group of patients, due to the high cost of therapy, but also the coexistence of health contraindications.

Since ancient times, people have used plants and preparations to accelerate the wound healing process. Often their use is based on tradition. Research in recent years has focused on the search for confirmation of the usefulness of traditional plant materials and also for new therapeutic tools used to accelerate the wound healing process. Bee honey, as well as other bee products (propolis and royal jelly), has already found application in medicine not only as immune system stimulants but also as chronic wound healing promoters [4]. The wound healing process can be sped up by the topical application of specific anti-inflammatory and antioxidant herbs. Among them, we can distinguish *Aloe vera*, *Bixa orellana*, as well as *Allium sativum*. The results suggest *A. vera* accelerates wound healing by promoting the proliferation and migration of fibroblasts and keratinocytes and by protecting keratinocytes from preservative-induced death [5]. For example, bixin extracted from *Bixa orellana* can be used to treat ulcers and other wounds due to its unique properties of inhibiting inflammation and accelerating collagen maturation and wound cramps [6]. Allicin, the active component of garlic, has been shown to have antimicrobial and anti-inflammatory properties; it speeds up collagen maturation in the skin tissues and acts as an antiseptic to prevent infection [7].

*Calendula officinalis* (also known as marigold) is a perennial herbaceous plant from the Asteraceae family, common in Central and Southern Europe, Western Asia, as well as the US [8]. According to the European Medicines Agency (EMA) report, alcoholic and oil extracts of *Calendulae flos* are used traditionally for skin inflammations, minor wounds, and mouth or throat inflammation [9]. The reducing inflammation and promoting wound healing by *Calendulae flos* extracts is mainly attributed to the presence of secondary metabolites: triterpenes [10,11,12,13], flavonoids [14,15], and carotenoids [16]. The positive effect on the wound healing process has been proven in numerous in vitro and in vivo studies, which were found to increase the proliferation and migration of human fibroblast and keratinocytes, stimulating angiogenesis and decrease collagen degradation after use of *Calendulae flos* extracts [14,15,16,17,18]. Furthermore, the *Calendulae flos* extract possesses some pharmacological features, which include antioxidant and anti-inflammatory action, as well as antibacterial, antifungal, and antiviral activity against various pathogens, e.g., *Staphylococcus aureus*, *Escherichia coli*, *Bacillus cereus,* and others [19,20,21].

Based on literature data, it was found that *C. officinalis* extract stimulated proliferation and migration of fibroblasts at low concentrations, e.g., 10 µg mL^−1^ enhanced cell numbers by 64.35%. The inhibition of proliferation showed that this effect is mainly due to the stimulation of migration [22]. Another work confirmed that the extracts from *C. officinalis* flowers influence the inflammatory phase of wound healing at the transcriptional and protein level by activating the transcription factor NF-κB and by increasing the amount of the chemokine IL-8 [16]. Influence on the extracts on the re-epithelialization phase of human immortalized keratinocytes in the scratch assay was only marginal. Additionally, the ethanolic extract enhanced the collagen content in the supernatant of human dermal fibroblasts in part by significantly reduced collagenase activity [16]. Another study conducted by Hormozi et al. showed the effect of *C. officinalis* extracts on the improvement of fibroblast function during the wound healing process as a result of the influence of the extract on expression TGFβ1 and bFGF cytokines in mouse embryo fibroblast cells [23].

The utility of the use of *Calendulae flos* extracts in wound healing was also confirmed in an in vivo assay. Using female Wistar rats, on the 8th day of the experiment, about 90% of the wound was healed in the extract-treated groups, whereas for the control healing it was only 50% [17].

Due to the high potential of *Calendulae flos* use in wound care, a number of clinical studies have been conducted. Eight clinical trials were conducted assessing the usefulness of *Calendulae flos* extracts in the wound healing process, including clinical trials evaluating acute and chronic wounds [24,25]. Findings from the studies on acute wound healing showed faster resolution of the inflammation phase with increased production of granulation tissue in the test groups treated with the extract [24,25]. Clinical control studies on venous ulcers demonstrated decreased ulcer surface area compared to controls [25]. Three randomized clinical trials assessed the potential for the extracts to prevent acute post-radiation dermatitis, with one study showing improvements compared to trolamine [25].

Although the adhesion is used for more than 30 centuries, the mechanisms that govern the adhesion between two materials remain complex. Due to the possibility of creating bioadhesive bonds between the polymer used in the drug form and the biological surfaces, it is possible to maintain the adhesive drug forms at the application site. Adhesion mechanisms can be described by the mechanical interlocking model, adsorption theory, the electrostatic attraction theory, as well as the diffusion or interdiffusion theory [26]. Bioadhesive polymers are of high molecular weight, biocompatible, biodegradable polymers used to join two surfaces where at least one of them is a living tissue.

Bioadhesive polymers are high molecular weight, biocompatible, biodegradable polymers used to join two surfaces where at least one of them is a living tissue. The purpose of using bioadhesive drug forms is to prolong the application time. Adhesive polymers can be divide into two groups: first generation: anionic (e.g., sodium alginate, Carbopol, and sodium hyaluronate), cationic (chitosan), and non-ionic (hydroxyethyl cellulose, hydroxypropyl cellulose, polyvinylpyrrolidone, and polyvinyl alcohol), and second generation: thiolated polymers (thiomers) of toiletginate-cysteine, chitosan-iminothiolane, chitosan-thiobutylamidine, chitosan-thioethylamidine, chitosan-thioglycolic acid, polyacrylic acid-cysteine, polyacrylic acid, homocysteine, polycarbethine acid cysteine, and lectin [27].

One of the most promising agents is chitosan. Chitosan is a biocompatible and biodegradable natural polymer. It is usually used in topical applications for wound healing, due to its antimicrobial activity, stimulating collagen synthesis, as well as properties that accelerate cell proliferation and the process of angiogenesis during wound healing [28,29,30,31,32,33]. Chitin and chitosan could enhance the migratory activity of the human umbilical cord vein derived endothelial cells [34]. Chitosan interacts with the fibroblast growth factor and enhances its bioavailability [35,36]. Chitosan conjugates with laminin are used to deliver keratinocytes in wounded skin [37], which could accelerate healing via re-epithelialization and regeneration of nerves in the dermis. Finally, chitosan and chitosan-based materials are able to generate hydrogels by the neutralization of NH_2_ groups to block the repulsion between chitosan chains thus resulting in the formation of hydrogels through hydrophobic interactions, chitosan crystallinity, and hydrogen bonding [26]. Additionally, chitosan is characterized by anti-infection, antioxidant, and remodeling features, which are required for polymers used in wound healing [38]. Due to those properties, chitin and its derivatives are used as scaffolds, hydrogels, or tissue adhesives for skin repair [39,40].

The purpose of the present study was to formulate a hydrogel delivery system containing *Calendulae flos* lyophilized extract with chitosan, with appropriate adhesive properties, ensuring extended contact with the inflammatory site and modified release of active substances for a long time.

## 2. Materials and Methods

### 2.1. Chemicals

Chlorogenic acid and narcissin (isorhamnetin-3-*O*-rutinoside), as analytical standards, were purchased from Roth GmbH (Karlsruhe, Germany) and Sigma-Aldrich Co. (St Louis, MO, USA), respectively. HPLC grade water, HPLC grade acetonitrile and acetate buffer were provided by the JT Baker–Avantor Performance Materials B.V. (Deventer, The Netherlands). Solvents used for determination of total flavonoid content were purchased from Avantor Performance Materials Poland S.A. (Gliwice, Poland). Chitosan (CS) 80/500 and 80/1000, with a deacetylation degree of 80%, were supplied by HMC^+^ GmbH (Halle, Germany). (Hydroxypropyl)methyl cellulose (HPMC viscosity 2.600–5.600 cP) and all other chemicals were from Sigma–Aldrich Chemical Co.

### 2.2. Preparation and Analysis of Calendulae flos Lyophilized Extract

#### 2.2.1. Extract Preparation

The flowers of *Calendula officinalis* L. were purchased from the “Kawon-Hurt”, Poland (Lot No. 992.2018). Total flavonoid content (TFC) in the plant material was determined according to European Pharmacopoeia (Ph. Eur.) 9th Edition, *Calendulae flos* monograph [41].

Three hundred grams of dried plant material of *Calendulae flos* were extracted three times with ethanol–water (7:3), each time for 30 min at 95 °C in a water bath. The obtained extracts were concentrated under the vacuum (BÜCHI Rotavapor R-210, Büchi Labortechnik GmbH, Essen, Germany) to a syrupy consistency, frozen, and then lyophilized (CHRIST 1-4 LSC, Osterode am Harz, Germany). The temperature on the freeze dryer shelf was increased and ranged from +15 to +20 °C, the temperature inside the product estimated −4 °C and condensation temperature was set to −48 °C. The freeze-drying was conducted at reduced pressure (1.030 mbar) by 48 h.

#### 2.2.2. Determination of Chlorogenic Acid and Narcissin Content

The presence and concentrations of active compounds in *Calendulae flos* lyophilized extract were determined by using the Ultra-High Performance Liquid Chromatography coupled with mass spectrometry (UHPLC-MS/MS) and the Ultra-High Performance Liquid Chromatography with diode array detector (UHPLC-DAD) methods. The identification of reference substances was carried out by using the UHPLC-MS/MS system (Nexera coupled with LCMS-8030, Shimadzu, Kyoto, Japan). While the determination of chlorogenic acid and narcissin in *Calendulae flos* lyophilized extract were carried out by using a highly sensitive ultra-high performance liquid chromatography (Dionex Thermoline Fisher Scientific, Waltham, MA, USA) equipped with a high-pressure pump (UltiMate 3000), an autosampler (UltiMate 3000), and a DAD detector (UltiMate 3000) with Chromeleon software version 7.0. The method was performed on an Acquity UPLC HSS T3 column (1.8 µm, 2.1 mm × 150 mm, Waters) at the column temperature of 24 °C. The elution was conducted using mobile phase A: HPLC grade water at pH 3.4 and mobile phase B: HPLC grade acetonitrile at pH 2.4. Each solvent was acidulated by using formic acid. The linear gradient was as follows: 3–13% B over 0.0–4.0 min, 13–17.5% B over 4.0–5.0 min, 17.5% B over 5.0–9.0 min, 17.5–24.5% B over 9.0–12.5 min, 24.5–30.0% B over 12.5–17.0 min, 30.0% B over 17.0–25.0 min, 3.0% B over 25.0 min, and 3.0% B over 25.0–30.0 min with a flow rate of 0.275 mL min^−1^. The chromatographic profile was recorded at 330 nm and the injection volume was 5.0 μL. The structural characterization of the compounds was made based on the mass fragmentation pattern obtained under positive ion mode. The most sensitive mass transition for [M + H^+^] ions was detected: *m*/*z* 771 to 625, 479, and 317 for typhaneoside and *m*/*z* 625 to 479 and 317 for narcissin.

The stock solutions of chlorogenic acid and narcissin were prepared by dissolving each compound in phosphate buffer (pH 5.5) at concentrations of 100.0 μg mL^−1^ and 250.0 μg mL^−1^, respectively. Working solutions were prepared by diluting stock solutions in phosphate buffers to achieve concentrations of chlorogenic acid and narcissin in the range 10.0–100.0 μg mL^−1^ and 10.0–250.0 μg mL^−1^, respectively. Each concentration level of analyzed compounds was injected six times into the UHPLC system for the construction of the calibration curves.

The method was validated according to the International Conference on Harmonization (ICH Q2 (R1)) guidelines for method validation [42]. The method was validated for linearity, precision, the limit of detection (LOD), and limit of quantification (LOQ).

The changes in the concentrations of chlorogenic acid and narcissin were also determined using chromatographic method during the dissolution and permeability studies.

#### 2.2.3. Scratch Wound Healing Assay

Normal human skin fibroblasts (CRL-1474) were purchased from the American Type Culture Collection (Manassas, VA, USA) and maintained in Dulbecco’s modified Eagle’s medium-high glucose (DMEM) supplemented with 10% fetal bovine serum (FBS), penicillin (100 U mL^−1^), and streptomycin (100 µg mL^−1^). The cells were cultured in a humidified environment at 5% CO_2_ and 37 °C. On the day of the experiment, cells were collected from subconfluent monolayers with trypsin/EDTA. The wound closure ability of human fibroblasts was assessed using a scratch assay. Fibroblasts were seeded into a 6-well plate at a concentration of 1 × 10^5^ cells mL^−1^ and the cells were cultivated in DMEM-high glucose supplemented with 10% FBS, penicillin (100 U mL^−1^) and streptomycin (100 µg mL^−1^). When the confluence of cells reached about 90%, the vertical linear wound (scratch) was generated in the monolayer with a sterile 200 µL pipette tip. Cells were washed three times with a phosphate buffer saline (PBS) to remove all cellular debris, and the fresh medium containing 2% FBS (control group) or medium with varying concentrations (i.e., 250 µg mL^−1^, 125 µg mL^−1^, 62.5 µg mL^−1^, and 31.25 µg mL^−1^) of *Calendulae flos* lyophilized extract was added to respective wells. Images of the scratch were taken after 0 h, 12 h, and 24 h using Olympus CKX53 microscope coupled with XM10 digital camera (Olympus). The experiments were performed at least in duplicate. Scratch area at the beginning of the experiment (0 h) was considered 100%. The open wound area was measured with the NIH ImageJ software (Bethesda, Rockville, MD, USA). Wound closure in % was calculated using the following formula:

(1) $$Closed\;wound\;area\;\left(\%\right)=\frac{open\;wound\;area\;at\;0\;h-open\;wound\;area\;at\;12\;h\;or\;24\;h}{open\;wound\;area\;at\;0\;h}\times 100\%$$

Results were expressed as the mean percentage of wound closure ± SD.

### 2.3. Preparation, Characterization, and Biological Activity of Chitosan Delivery System with Calendulae flos Lyophilized Extract

#### 2.3.1. Chitosan Binary System

The *Calendulae flos* lyophilized extract was mixed in a mortar together with two types of chitosan with a degree of deacetylation of 80% and different viscosity (500 mPas and 1000 mPas), in weight ratio 1:1 (*w*/*w*) and 1:5 (*w*/*w*) for 45 min in order to obtain a chitosan delivery system.

#### 2.3.2. Dissolution Studies of Chitosan Delivery Systems

Dissolution studies of active compounds from chitosan delivery systems were carried out by using a standard paddle Agilent 708-DS Dissolution Apparatus (Agilent Technologies, Santa Clara, CA, USA) with a 150-mL dissolution medium at 32 ± 0.5 °C and 50 rpm for 480 min. In this order, samples of chitosan delivery systems were weighed into gelatine capsules and then placed in the spring in order to prevent flotation of the capsule on the surface of the liquid. As dissolution media, phosphate buffer at pH 5.5, simulating skin pH, was used. At appropriate time intervals, dissolution samples (1.0 mL) were collected with the replacement of equal volumes of temperature-equilibrated media and filtered through a 0.45 μm membrane filter. The active compounds concentration was measured by using the UHPLC-DAD method. The similarity of the dissolution percentage of active compounds from chitosan delivery systems was established based on *f*_1_ and *f*_2_ parameters and was defined by the following equation:

(2) $$f_{1}=\frac{\sum_{j=1}^{n}\left|R_{j}-T_{j}\right|}{\sum_{j=1}^{n}R_{j}}\times 100$$

(3) $$f_{2}=50\times \log\left({\left(1+\left(\frac{1}{n}\right)\sum_{j=1}^{n}{\left|R_{j}-T_{j}\right|}^{2}\right)}^{-\frac{1}{2}}\times 100\right)$$

in which *n* is the number of withdrawal points, *R*_j_ is the percentage dissolved of reference at time point *t*, and *T*_j_ is the percentage dissolved by test at time point *t*. The *f*_1_ value close to 0, and *f*_2_ value close to 100 indicate profile similarity [43].

#### 2.3.3. Permeability Studies of Chitosan Delivery Systems

Permeability through artificial biological membranes of active compounds from *Calendulae flos* lyophilized extract and from chitosan delivery systems was investigated by using the Skin PAMPA method (skin parallel artificial membrane permeability assay). The Skin PAMPA model consists of a two-chamber PAMPA sandwich composed of two 96-well plates. The top plate contains the lipid-impregnated skin-mimetic membrane. Before use, pre-coated Skin PAMPA™ sandwich plates (Pion Inc.) were hydrated overnight by placing 200 μL of the hydration solution in each well (Hydration Solution, Pion Inc.). Each experiment was repeated at least three times, using six replicates on each plate. The amount of permeated active compounds was determined using the UHPLC method.

The apparent permeability coefficients (*P_app_*) were calculated from the following equation:

(4) $$P_{app}=\frac{-ln\left(1-\frac{C_{A}}{C_{equilibrium}}\right)}{S\times \left(\frac{1}{V_{D}}+\frac{1}{V_{A}}\right)\times t}$$

where *V_D_*—donor volume, *V_A_*—acceptor volume, *C_equilibrium_*—equilibrium concentration $C_{equilibrium}=\frac{C_{D}\times V_{D}+C_{A}\times V_{A}}{V_{D}+V_{A}}$, *C_D_*—donor concentration, *C_A_*—acceptor concentration, *S*—membrane area, and *t*—incubation time (in seconds).

To verify that *P_app_* determined for permeability was statistically different, an ANOVA test was used. Compounds with *P_app_* < 1 × 10^−6^ cm s^−1^ are classified as low-permeable and those with *P_app_* > 1 × 10^−6^ cm s^−1^ as high-permeable compounds [44].

#### 2.3.4. Anti-Hyaluronidase Activity

The inhibition of hyaluronidase was determined by a turbidimetric method described by Studzińska-Sroka et al. [45]. The chitosan delivery systems in weight ratio 1:1 and *Calendulae flos* lyophilized extract were shaken (150 r min^−1^) with acetate buffer on an incubated shaker (Thermo Scientific MaxQ 4450, MA, USA) for 1 h at 37 °C and then centrifuged at 9000 r min^−1^ for 10 min (Nüve NF800, Ankara, Turkey) to produce a clear supernatant. The final concentrations were 15–120 µg mL^−1^ and 500–750 µg mL^−1^ for chitosan delivery systems and *Calendulae flos* lyophilized extract, respectively. Quercetin (final concentrations of 2.0–4.0 mg mL^−1^) was used as a positive control. The IC_50_ values were calculated with OriginPro 9 software using linear regression.

#### 2.3.5. Antimicrobial Activity

The chitosan delivery systems with *Calendulae flos* lyophilized extract were dissolved in 1.0 mL of dimethyl sulfoxide (DMSO). From the obtained stock solutions (at a concentration of 100 mg mL^−1^), a series of dilutions in the concentration range 0.5–15.0 mg mL^−1^ were prepared in the antibiotic broth medium (Merck). For each 1 mL of dilution of the extract or fraction, 0.1 mL of 18-h-old liquid culture of standard strains diluted 1:10,000 in the same medium was added (the number of cells added was approximately 10^3^ in 0.1 mL). The samples were incubated at 37 °C for 24 h. After this time, all dilutions were inoculated on solid antibiotic agar. After a further 24 h, the lowest concentration of sample dilutions, which completely inhibited the reference strain growth was marked as the minimal bactericidal concentration (MBC) [46,47]. The study evaluated the antibacterial activity of the *Calendulae flos* lyophilized extract and chitosan delivery systems on selected bacterial strains (*Staphylococcus aureus, Staphylococcus epidermidis, Enterococcus faecalis, Enterococcus faecium, Streptococcus pyogenes, Escherichia coli, Pseudomonas aeruginosa,* and *Propionibacterium acnes)* and fungi (*Candida albicans*).

### 2.4. Formulation of the Hydrogel Topical Pharmaceutical Dosage Containing Chitosan Delivery System with Calendulae flos Lyophilized Extract

Hydrogel formulations were prepared by dispersing hypromellose (HPMC, 2% or 3%, *w*/*w*) in ultrapure water by continuous stirring at 60 °C for 1 h. Than 3% and 10% *w/w Calendulae flos* lyophilized extract or chitosan delivery systems in the weight ratio 1:1 were added to the polymeric solution (Table 1) and stirred gently with a magnetic stirrer until the homogeneous gel was formed. Then, the system was cooled down under stirring and allowed to equilibrate for 24 h at room temperature to assure the complete hydration of HPMC.

#### 2.4.1. Microscopic Structure of Hydrogels by Scanning Electron Microscopy (SEM)

Morphology and microstructure of hydrogels was studied by classical scanning electron microscopy Hitachi TM3000. The hydrogel was freeze-died for 24 h at −55 °C at 6.5 hPa before measurements, using lyophilizer Heto PowerDry PL3000, Thermo Scientific. Samples for SEM were sputtered with gold to improve conductivity and obtain better image quality.

#### 2.4.2. Rheological Analysis

The rheological measurements were performed on a HAAKE^TM^ RheoStress1 (Thermo Electron Corp., Waltham, MA) rotational rheometer. The temperature was controlled by HAAKE^TM^ DC30 thermostat with a recirculating water bath. The samples were tested with the titanium plate–plate geometry (35 mm). The samples were placed on the lower plate with a spatula. The measuring gap was set at 1.0 mm. After lowering the upper plate, the excess of the sample was gently removed by a spatula with special care to avoid any unwanted shearing. The temperature during measurements was set at 32.0 ± 0.5 °C. The data obtained were analyzed and calculated on HAAKE^TM^ RheoWinTM Data Manager Software (Thermo Electron Corp., Waltham, MA, USA). Each measurement was carried out using a fresh sample. Each measurement was conducted in triplicate using fresh samples, and the mean values of the various parameters were reported.

Steady shear experiments involved flow curves (CR—controlled rate) and stress ramp test (CS—controlled stress). The flow curves were plotted as the dependence of the shear stress (*τ*) on the shear rate ($\dot{\gamma }$). The shear rate ranged from 0 to 10.0 s^−1^ (increasing ramp) and back (decreasing ramp). The time of each ramp was 30 s. No interval between the ramps was set. While the measurements of stress ramp tests were performed under changing shear stress in the range of 1.0–200.0 Pa. The time of the assay was 60.0 s. The results were plotted as the dependence of strain vs. shear stress, both on a logarithmic scale.

Oscillatory shear measurements involved stress sweep (SS) and frequency sweep (FS). During stress sweeping, the samples were exposed to the increasing oscillatory stress (1.0–200.0 Pa) at a constant frequency (f = 1.0 Hz). The shear stress (*τ*), storage modulus (*G*′), and loss modulus (*G*″) values were plotted on a logarithmic scale. While for frequency sweep the samples were exposed to increasing frequency (f = 1.0–100.0 Hz) at constant oscillatory stress (2.0 Pa). The results are presented on a logarithmic scale as the dependence of *G*′, *G*″ on f.

#### 2.4.3. Drug Release Profiles

The in vitro release experiments were performed for the HPMC hydrogels with the use of vertical Franz cells (PermeGear Hellertown, Pennsylvania, USA), each containing 8 mL of acceptor solution (phosphate buffer; pH = 5.5). The cells were equipped with regenerated cellulose membranes (Visking^®^ dialysis tubing, SERVA Electrophoresis GmbH, Heidelberg, Germany) with pore diameter ca. 25Å. The membranes were kept immersed in the acceptor fluid at 32.0 ± 0.5 °C for 24 h before the experiment. Gel samples (1.0 mL) were placed at the donor compartment and spread evenly on the surface of the artificial membrane. The effective diffusion area of the employed cells was 0.999 cm^2^. The receptor fluid during the test was stirred at 200 rpm, and its temperature was set at 32.0 ± 0.5 °C. The samples (1.0 mL) were taken from the acceptor compartment after every 30–420 min and replaced immediately with an equal volume of fresh acceptor fluid. The chlorogenic acid and the narcissin concentrations in the collected samples were determined with the UHPLC-DAD method described above.

#### 2.4.4. Antimicrobial Activity

Indicator microorganisms were transferred to test tubes containing Mueller–Hinton medium. They were cultured at 37 °C for 24 h. Next, the liquefied agar medium was inoculated with 10% (*v*/*v*) 24 h indicator culture and poured into Petri dishes to obtain a distinct confluent layer. After solidification of the broth medium inoculated with the indicator microorganisms (*Staphylococcus aureus, Staphylococcus epidermidis, Enterococcus faecalis, Enterococcus faecium, Streptococcus pyogenes, Escherichia coli, Pseudomonas aeruginosa, Propionibacterium acnes,* and *Candida albicans)*, wells were made with a cork borer. Each well was supplemented with 150 mg of hydrogel formulations F5-F8. Next, the diameters of the indicator bacteria growth inhibition or reduction were measured. The inhibition of the growth of the indicator microorganisms was manifested by complete lightening around the place where the hydrogel was transferred. It indicated the bactericidal activity of the bacterial strain. Bacteriostatic properties were determined by measuring the diameter of the growth inhibition zone (indicator strain growth limitation).

## 3. Results and Discussion

Alcoholic and oil *Calendulae flos* extracts are traditionally externally used for the treatment of various skin injuries, e.g., bruises, frostbite, burns, leg ulcers, as well as inflammation of the mouth, throat or vagina mucous membranes due to their anti-inflammatory properties, antibacterial, angiogenic, and fibroblastic. Semi-solid preparations containing *Calendulae flos* extracts are in the form of ointments; therefore their use is limited in the treatment of chronic wounds.

Therefore, a hypothesis was built that combines the valuable medicinal properties of *C. officinalis* with an appropriate delivery system will create a new, valuable solution in the treatment of chronic wounds. Chitosan was chosen as a suitable carrier. It ensures the adhesion of active substances at the site of application and a prolonged release of active substances. It additionally has an antimicrobial effect, accelerating cell proliferation, and modulating the process of angiogenesis. Therefore, it was chosen to prepare delivery systems containing *Calendulae flos* lyophilized extract. The synergism of the combination of *Calendulae flos* lyophilized extract and chitosan will be made up of stronger biological activity as well as better adhesive properties.

The first stage of experimental work was to prepare freeze-dried hydroalcoholic *Calendulae flos* extract. Raw plant material was *Calendulae flos.* It was obtained from the “Kawon-Hurt” (number of GMP certificate: WTC/0074_01_01/77). The plant material used for these studies met the pharmacopoeial requirements in accordance with the total flavonoid content (TFC). The TFC of *Calendulae flos* was 0.95% ± 0.04%. According to Ph. Eur. 9th Edition, the dried flowers of *Calendula officinalis* should have a minimum of 0.4% of flavonoids, expressed as hyperoside [41].

**Figure 1:**
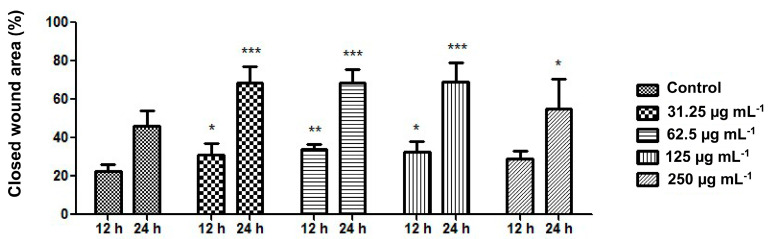
Effect of the different concentrations of *Calendulae flos* lyophilized extracts on the closed wound area. Results are expressed as means ± SD. Statistical significance was designated at (*) *P* < 0.05; (**) *P* < 0.01; (***) *P* < 0.001 (versus control at respective time point) using ANOVA followed by Bonferroni’s post hoc test.

**Figure 2:**
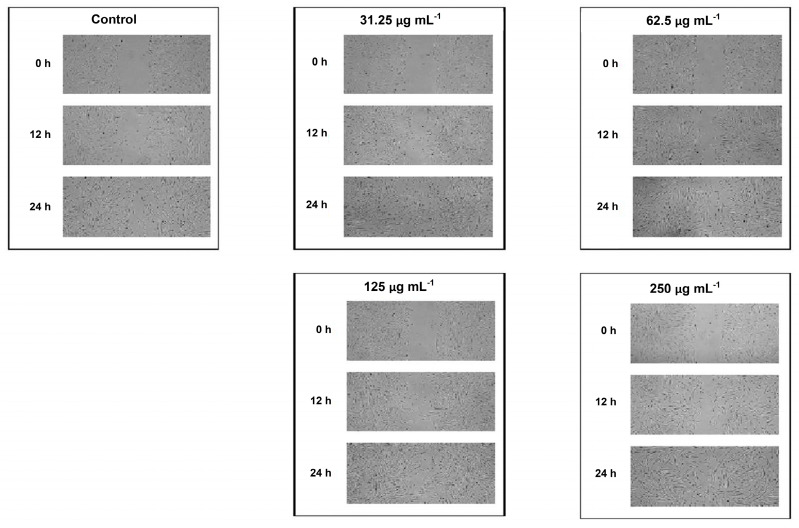
Effect of the different concentrations of *Calendulae flos* lyophilized extract on fibroblast cell migration.

Next, ethanol–water (7:3) extract of plant material of *Calendulae flos* was lyophilized. The extract was standardized for the content of chlorogenic acid and narcissin; therefore the biological effect of all compounds present in the extract (e.g., carotenoids, triterpenes, and phenolic compounds) will be tested during a scratch test. A scratch test is usually created in a cell line to observe the wound healing process, in which the cells polarize towards the wound, initiate protrusion, migrate, and close the wound [22,48]. As reported in Figure 1 and Figure 2, each of the tested concentrations of *Calendulae flos* lyophilized extract was able to increase the wound-healing rate compared to the untreated control. After 12 h and 24 h of the treatment, a significant increase the wound-healing rate by 31%, 34%, and 33% and 68%, 68%, and 69% was observed for concentrations of 31.25, 62.5, and 125.0 µg mL^−1^ respectively, compared to the untreated control (23% and 46%). The highest concentration (250 µg mL^−1^) demonstrated the weakest effect on the migration of fibroblasts, and increasing the wound healing was 29% after 12 h and 55% after 24 h.

The downward trend observed for the concentration of 250 μg mL^−1^ is consistent with the results obtained by other authors. For example, Hormozi et al. examined the wound healing effect of *C. officinalis* extract administered at the following concentrations: 5, 10, 20, 40, and 50 µg mL^−1^. Their studies confirmed that concentrations of 5 μg mL^−1^ and 10 μg mL^−1^ were more suitable for cell proliferation of fibroblasts [23]. Moreover, Fronza et al. showed that both hexane and ethanolic extracts from *C. officinalis* stimulated proliferation and migration of fibroblasts at low concentrations (i.e., 1 and 10 µg mL^−1^) [22]. This phenomenon may result from the fact that *C. officinalis* extract stimulates the proliferation of fibroblasts via increased expression of growth factor TGF-β1 [23]. However, only low concentrations of TGF-β1 activate additional signaling pathway (p38 MAPK), which is crucial to the fibroblasts migration [49]. Additionally, a reduced wound healing activity observed for higher concentration of *C. officinalis* extract might simply result from the overload of cells with different components of the extract that eventually causes reduced proliferation and migration of cells.

As a result, the scratch test analysis confirmed the maintenance of the biological activity of the *Calendulae flos* extract after freeze-drying in relation to its effect on the proliferation and migration of fibroblasts [15,16]. It can, therefore, be suggested that there have been no changes in the composition of critical groups of compounds that correspond to biological activity. Analysis of chlorogenic acid and narcissin indicated that the weakest effect was observed at their highest concentrations. A similar dose-dependent trend was observed by Dinda et al., who found the highest migration of fibroblast cells after 24 h was after treatment with hydroethanolic *Calendulae flos* extract at a concentration of 100 μg mL^−1^ [15]. Considering that lower doses of chlorogenic acid and narcissin may have a stronger therapeutic effect, it is justified to design a system of delivery of active compounds that will release lower doses over time. The development of the form of *Calendulae flos* lyophilized extract excludes the possibility of changing the content of key active compounds, which is an advantage over easily evaporating extracts, especially with alcohol content. This approach also ensures that the amounts of chlorogenic acid and narcissin released will be the same in time.

**Figure 3:**
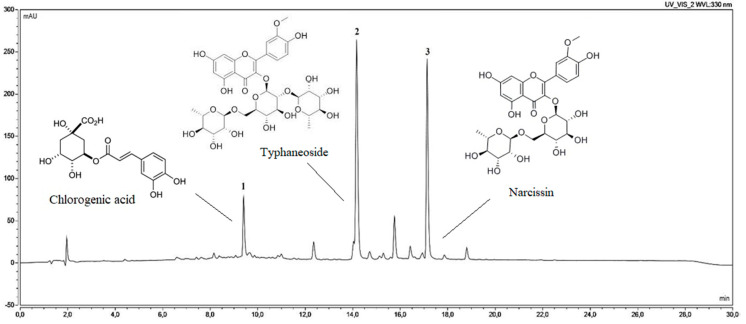
The chromatogram of 1—chlorogenic acid, 2—typhaneoside, and 3—narcissin, presented in the *Calendulae flos* lyophilized extract.

The already mentioned standardization of *Calendulae flos* lyophilized extract using the UHPLC-DAD method was carried out in relation to the determination of standards: the chlorogenic acid and the narcissin. Concentration changes of those compounds were studied during also dissolution and permeability studies of standards from chitosan delivery system, as well as release studies of standards from hydrogel forms. Those analyses were successfully performed with ultra-high performance liquid chromatography on a C_18_ column with gradient elution of water (pH 3.4) and acetonitrile (pH 2.4), at the flow rate of 0.275 mL min^−1^, and column temperature at 24 °C. As shown in Figure 3, on the UHPLC chromatogram three main peaks were presented. The comparison of retention times (9.45, 14.15, and 17.12 min) with one of the reference standards allows us to identify the order as follows: 1—chlorogenic acid, 3—narcissin. The identification of the second peak was also conducted based on MS/MS spectra, and it was: 2—typhaneoside (Table 2). Our results were compatible with literature data [50]. Using the linearity equations of reference substances, it was possible to determine the content of standards in the *Calendulae flos* lyophilized extract. The chlorogenic acid content was 4.22 ± 0.05 mg per 1 g of lyophilized extract, while narcissin content was 64.63 ± 0.39 mg per 1 g of lyophilized extract.

Validation of the UHPLC-DAD (Table S1) method followed the protocol issued by ICH Q2 (R1) for linearity, precision, limits of detection, and quantification of analytical standards was collected in the Supplementary Materials.

**Figure 4:**
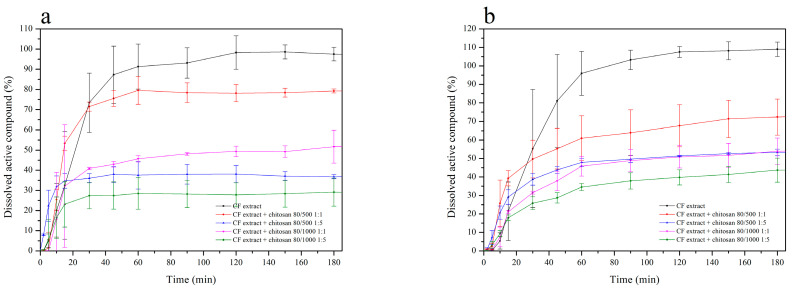
Release profiles of the chlorogenic acid (**a**) and the narcissin (**b**) from the *Calendulae flos* lyophilized extract and prepared chitosan delivery systems.

Several reports indicate chitosan as a carrier with significant adhesive properties [27,51,52]. It was confirmed that systems of plants extracts or polyphenolic compounds with chitosan had shown a synergy effect, e.g., quercetin [53], rutin [54], curcumin [55,56], rosmarinic acid [57], propolis [58], *Melilotus officinalis* [59], *Passiflora edulis* [60], and *Musa cavendishii* [61]. Concerning the possibility of achieving synergy of biological activity and ensuring prolonged contact of *Calendulae flos* lyophilized extract with the wound, chitosan delivery systems were obtained. The effects of chitosan viscosity and the ratio between chitosan and *Calendulae flos* lyophilized extract were analyzed as system variables. In our study, the *Calendulae flos* lyophilized extract chitosan was prepared by mixing in a mortar with two types of chitosan in a degree of deacetylation of 80% and different viscosity (500 mPas and 1000 mPas), in weight ratio 1:1 (*w*/*w*) and 1:5 (*w*/*w*). The effect of various concentrations and viscosities of chitosan on the dissolution rate profiles of the chlorogenic acid and the narcissin from chitosan delivery systems with *Calendulae flos* lyophilized extract was evaluated. The differences in the dissolution profiles of *Calendulae flos* lyophilized extract and chitosan delivery system 80/500 in weight ratio 1:1 (*w*/*w*) is shown in Figure S1 (Supplementary Materials). All tested chitosan delivery systems reached a plateau at the 180th minute, but the study was conducted until 480 min to confirm the prolonged release. As shown in Figure 4 the release rate of the chlorogenic acid and the narcissin from chitosan delivery systems increased gradually with time (mainly during the first 60 min of the study) and decreased with increasing amount and viscosity of chitosan in all tested systems (*Calendulae flos* lyophilized extract-chitosan 80/500 (1:1 (*w*/*w*)) > *Calendulae flos* lyophilized extract-chitosan 80/1000 (1:1 (*w*/*w*)) > *Calendulae flos* lyophilized extract-chitosan 80/500 (1:5 (*w*/*w*)) > *Calendulae flos* lyophilized extract-chitosan 80/1000 (1:5 (*w*/*w*)), compared to the *Calendulae flos* lyophilized extract without chitosan. It is suspected that the initial rapid release of active compounds may be the result of incomplete adsorption or weak binding of part of the extract to the surface of chitosan nanoparticles [62]. The effectiveness of the lower concentrations of the *Calendulae flos* lyophilized extract in a scratch wound healing assay seems to justify loading the extract with chitosan and obtaining a prolonged release of the active compounds.

The skin is an accessible and convenient place for drug administration, especially when the action of active compounds is planned at the site of application and does not require a systemic effect [63]. However, we must remember that for biologically active compounds to modify inflammatory signaling pathways; they must penetrate not only through the epidermis into the deeper skin layers; therefore our research also involved an assay of penetration of the chlorogenic acid and the narcissin through biological membranes that stimulate the skin barrier. Using the Skin PAMPA model, it was possible to study the permeability of the chlorogenic acid and the narcissin by passive diffusion. As a result of a 5-h incubation, the time needed to release the active substances from the chitosan delivery systems with *Calendulae flos* lyophilized extract in weight ratio 1:1 (*w*/*w*) and allowing of the substance permeation through a barrier, it was possible to determine the concentrations of standards and to calculate the apparent permeability values Papp for the chlorogenic acid ((2.42 ± 1.18) × 10^−6^ cm s^−1^) and the narcissin ((2.35 ± 1.46) × 10^−6^ cm s^−1^). Those values were higher than 1 × 10^−6^ cm s^−1^, so it was possible to classify standards as high-permeable compounds [44]. Then permeability of those standards from the *Calendulae flos* lyophilized extract, as well as its chitosan delivery systems were also investigated (Table 3). Values *P_app_* for the chlorogenic acid and narcissin from *Calendulae flos* lyophilized extract and its chitosan delivery systems were not significantly different from those obtained for free standards. There is no detailed literature data about the mechanism of skin transporters for active compounds from *Calendulae flos*. In the case of in vitro absorption studies and gut absorption, data indicate passive diffusion as the primary transport mechanism [64,65,66]. Similarly, most phenolics, e.g., narcissin and chlorogenic acid, are absorbed via passive diffusion [67,68]. Moreover, chitosan viscosity did not influence the chlorogenic acid and the narcissin permeation, and a mechanism of chitosan interaction with skin is not proven [39].

**Figure 5:**
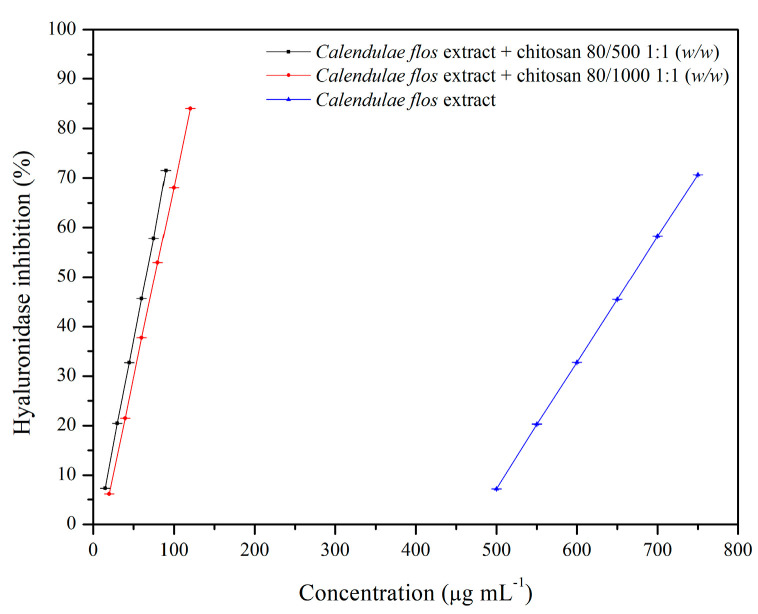
Anti-hyaluronidase activity of the *Calendulae flos* lyophilized extract and its chitosan delivery systems expressed as IC_50_ (50% inhibition of enzyme activity).

The biological activities of the chitosan delivery systems were tested using in vitro models, assessing the possibilities of hyaluronidase inhibition and the potential of antimicrobial activity. Hyaluronic acid plays a significant role in the wound healing process, including hydration, anti-inflammation, and stimulation of cellular migration; therefore, this model was chosen to evaluate the anti-inflammatory effect of the system [69]. The degradation of products, the low molecular weight of hyaluronic acid, exhibits pro-angiogenic and pro-inflammatory properties [70]. There are no reports found in the literature regarding the effect of *Calendula flos* extracts on hyaluronidase inhibition. The stronger anti-hyaluronidase activity of the *Calendulae flos* lyophilized extract (IC_50_ = 667.94 µg mL^−1^) than the positive control quercetin (IC_50_ = 3.22 mg mL^−1^) is an effect of the synergistic action of many groups of active compounds such as triterpenoids [71], flavonoids (e.g., quercetin, rutin, and isorhamnetin), and phenolic acids (especially chlorogenic acid), which are well-known as good anti-inflammatory agents [72,73,74]. The strongest activity was observed for chitosan delivery systems that were approximately ten times more potent than *Calendulae flos* lyophilized extract alone (Figure 5). Chitosan viscosity did not significantly affect hyaluronidase inhibitory activity, and there were IC_50_ = 65.40 µg mL^−1^ for chitosan 80/500 and IC_50_ = 76.11 µg mL^−1^ for chitosan 80/1000.

Concerning the significant increase of the anti-inflammatory effect of the chitosan delivery systems of the *Calendulae flos* lyophilized extract, the antimicrobial potential of the prepared chitosan systems was also examined. A Gram-positive (mostly *Staphylococcus aureus*) and Gram-negative (mostly *Pseudomonas aeruginosa* and *Escherichia coli*) species are the most commonly isolated from chronic wounds. It also was found that in many cases, burn wounds are co-infected by *Candida* sp. [75]. *Calendulae flos* extracts have well documented antimicrobial activity [19,20,21], especially polyphenols and essential oil, are useful in wound healing [76,77]. As shown in Table 4, it is noteworthy that the combination of the *Calendulae flos* lyophilized extract with chitosan did not exhibit a lower MBC value, in particular against pathogens responsible for wound infections. In the case of *Candida albicans* infection, significant decreases in MBC were also observed. Decreases of MBC values were mainly found in the case of chitosan 80/1000 delivery system 1:1 (*w*/*w*; from 64 to 8 mg L^−1^), chitosan 80/500 delivery system 1:1 (*w*/*w*; from 64 to 32 mg L^−1^), and chitosan delivery 80/500 system 1:5 (*w*/*w*; from 64 to 32 mg L^−1^). In the case of *Propionibacterium acnes*, these changes were also beneficial. Data indicates the synergism of bactericidal activity of chitosan and the *Calendulae flos* lyophilized extract against the anaerobic pathogen, which often infects the skin [78,79].

In the last stage of the work, optimization of obtaining a topical pharmaceutical form containing the *Calendulae flos* lyophilized extract with chitosan 80/500 1:1 (*w*/*w*) and 80/1000 1:1 (*w*/*w*) was carried out to obtain appropriate adhesive properties for the supporting strategy for wound healing applications. The chitosan delivery systems 80/500 1:1 (*w*/*w*) and 80/1000 1:1 (*w*/*w*) were selected based on the most favorable release profile, the possibility of hyaluronidase inhibition and antimicrobial activity data. Hydrogels were selected as a pharmaceutical dosage form with controlled release of active substances. They can be considered as ideal topical preparations for the treatment of chronic wounds due to their ability to cleanse the wound by absorbing exudate along with impurities, maintaining adequate humidity on the wound surface, and controlling the penetration of water vapor and oxygen [80]. Furthermore, chitosan-based hydrogels are used as delivery systems for the controlled release of therapeutic ingredients [81], due to its adhesive and biodegradable properties, as well as the ability to open tight epithelial junctions transiently [82]. As a base of hydrogel hydroxypropyl methylcellulose (HPMC) was selected. HPMC is used as a thickening agent, binder, film former, and hydrophilic matrix material. HPMC is a popular matrix material in controlled delivery systems, and HPMC matrices show sustained release patterns by two mechanisms, i.e., diffusion and erosion of the gel layer. The viscosity of the polymer affects the diffusion pathway [83]. In cases where biodegradability of a hydrogel is required or recommended, HPMC is appealing to hydrogel precursor materials, due to their low cost, the large availability, and biocompatibility of cellulose [80]. Moreover, HPMC, in a concentration of 2–3%, showed the consistency and viscosity required for topical administration [84]. HPMC-based hydrogels are widely used with herbal preparation such as *Aloe vera* [85] as well as an herbal gel containing *Melissa officinalis*, *Rhus coriaria*, *Glycyrrhiza glabra*, *Rosmarinus officinalis*, and *Pelargonium roseum* [86].

**Figure 6:**
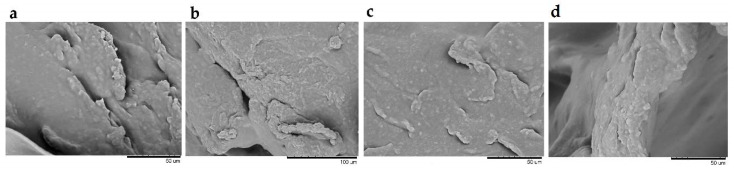
Scanning electron microscopy images of hydrogels F5 (**a**), F6 (**b**), F7 (**c**), and F8 (**d**); a, c, d—magnification ×1500, b—magnification ×1000.

To determine the microstructure and surface morphology of the hydrogel formulations, SEM images were taken. According to the literature, the HPMC-based hydrogels present a porous inner structure and homogeneous open pores [87]. As seen on the SEM images (Figure 6), prepared hydrogels have an uneven and coarse surface structure. Changes in the structure may be the result of a freeze-drying process that affects the morphology and pore size of hydrogels [88], as well as the presence of chitosan, which is characterized by nonporous and relatively smooth surface morphologies [89,90].

Fresh-prepared HPMC-based hydrogels have undergone rheological tests, divided into two parts, steady shear and oscillatory shear measurements. In the first case, the samples were tested at a controlled rate (CR) and controlled stress (CS) modes. The results of CR assays were plotted as standard flow curves (Figure S2) and are presented in the Supplementary Materials. The shapes of the plots suggest that all the formulations revealed non-Newtonian, pseudoplastic (shear thinning) properties. The viscosity of such materials decreases with the shear rate increase. In order to characterize the gels more precisely, the curves were fitted with the Ostwald de Waele model:

(5) $$\tau =K\cdot {\dot{\gamma }}^{n}$$

where *n* is a power-law index (fluidity) and depicts how far from Newtonian behavior (*n* = 1) is given material. The values of *n* < 1 mean that the sample is shear thinning, whereas *n* > 1 reflects the dilatant (shear thickening) nature. The *n* parameter is also defined as the rate of gel structure change upon the shear rate. The *K* value describes the consistency coefficient and is equal to the shear stress at a shear rate of 1.0 s^−1^. The data presented in Table 5 show that the plots were adjusted to the Ostwald de Waele model with good correlation and reproducibility. The results indicate that among all samples, the increasing amount of HPMC contributed to the increase of pseudoplasticity. Moreover, it was observed that the increase of *Calendulae flos* lyophilized extract percentage also entailed more shear thinning behavior. Similarly and not surprisingly, the consistency was increasing with the amount of HPMC and *Calendulae flos* lyophilized extract. Thixotropy calculations showed that the structure recovery of the samples was very fast after thinning.

The CS ramp test was performed to calculate the yield stress of the samples, which is defined as a critical force that has to be applied to initiate the flow and damage the polymer network. Yield stress defines many aspects of product processing, handling, storage, and performance. The proper yield stress value correlates with the appearance or application behavior and determines the conditions during production mixing, pumping, extrusion or container filling, etc. The plots of deformation vs. shear stress are presented on a logarithmic scale in Figure S2. As can be observed, the plots revealed none or very little inflection; therefore it was not possible to determine the yield values for the samples. Such behavior indicates that the elastic structure of the samples was very weak or negligible; thus, the materials possessed no apparent yield stress and started to flow in the moment of the force application.

During oscillatory shear measurements, the samples were conducted to changing dynamic stress and dynamic frequency. The oscillatory stress sweeping was used to monitor the values of storage (*G*′) and loss moduli (*G*″) upon the increasing oscillatory stress. The angular frequency of the oscillation was constant during the experiments (1 Hz = 6.2832 rad s^−1^). The logarithmic plots presented in Figure S2 confirmed the observations from previous tests. The samples revealed no elastic behavior, while during the whole measurement *G*″ predominated *G*′ and were liquid-like. However, it can be assumed that the larger the amount of HPMC or *Calendulae flos* lyophilized extract, the smaller the value of tan δ, which means the increase of the elastic response. The frequency sweeping of the samples showed that both the moduli increased with f. At certain *f* values, the crossover points were observed. Such behavior is typical for entangled polymer networks, where the polymer concentration exceeds the critical overlap concentration, which results in bridging connections between the chains. It can be assumed that no behavior typical for weak gels was revealed by the samples.

To sum up, based on flow curves, it is possible to observe that all hydrogels were characterized by a low flow limit, they did not actually have it. This is important because only a gel with a low flow limit can easily be spread on diseased tissue. A condition for high pharmaceutical availability of the substance from medicinal products is not only a low viscosity gel but also a small area of the hysteresis loop.

The release kinetics of the chlorogenic acid and the narcissin from HPMC-based hydrogel containing chitosan delivery system with *Calendulae flos* lyophilized extract was determined with the use of vertical Franz diffusion cells equipped with a regenerated cellulose artificial membrane. To find the appropriate HPMC-based matrix (2% or 3%) as well as the amount of the *Calendulae flos* lyophilized extract (3% or 10%), formulation F1-F4 was chosen for dissolution studies. Moreover, the type of chitosan (80/500 and 80/1000 1:1 (*w*/*w*)) was also considered (F7 and F9).

**Figure 7:**
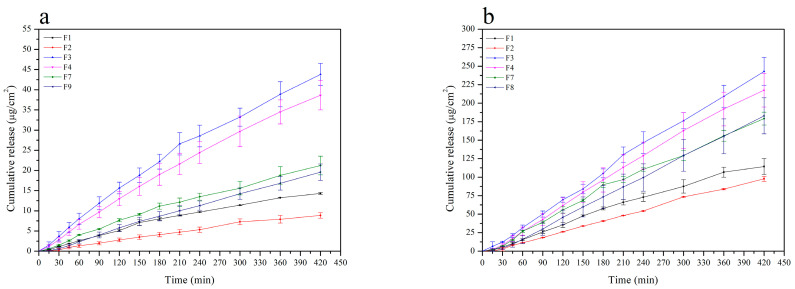
Dissolution profiles of chlorogenic acid (**a**) and narcissin (**b**) from HPMC-based hydrogel with chitosan delivery system with *Calendulae flos* lyophilized extract at pH = 5.5, where F1—2% HPMC-based hydrogel with 3% of *Calendulae flos* lyophilized extract, F2—3% HPMC-based hydrogel with 3% of *Calendulae flos* lyophilized extract, F3—2% HPMC-based hydrogel with 10% of *Calendulae flos* lyophilized extract, F4—3% HPMC-based hydrogel with 10% of *Calendulae flos* lyophilized extract, F7—2% HPMC-based hydrogel with 10% of *Calendulae flos* lyophilized extract + chitosan 80/500, and F9—2% HPMC-based hydrogel with 10% of *Calendulae flos* lyophilized extract + chitosan 80/1000.

The obtained results are shown in the form of release profiles in Figure 7 and Table 6 as the dependence of cumulative release (µg/cm^2^) vs. time (min). As can be observed, the shapes of the profiles indicate that in the case of every formulation, the process could be fitted to and characterized by the zero-order kinetics model with a satisfactory correlation. Two main parameters were calculated, namely the drug flux (*Jss*) and the average cumulative amount per area. According to a one way ANOVA test and post-Scheffe’s analysis, the release of the chlorogenic acid revealed no distinct differences for formulations containing 3% of the extract, and the increase of the HPMC amount had a very slight but noticeable impact on lowering of the drug flux. The main reason for this was attributed to the increase of viscosity and, therefore, less efficient diffusion of the active compound among the polymer network to the membrane. The thicker vehicle was also more resistant to the penetration of the acceptor fluid. It was also observed that the most efficient release occurred from the formulations that contained 10% of the extract and no addition of chitosan. The addition of chitosan resulted in a decrease of permeation, but changing the type of chitosan had no apparent influence on the process. Very similar effects concerning the release parameters were observed in the case of narcissin. The drug flux was significantly higher in every case, but the dependencies according to the composition of the formulations, were following the same pattern as for the chlorogenic acid.

**Figure 8:**
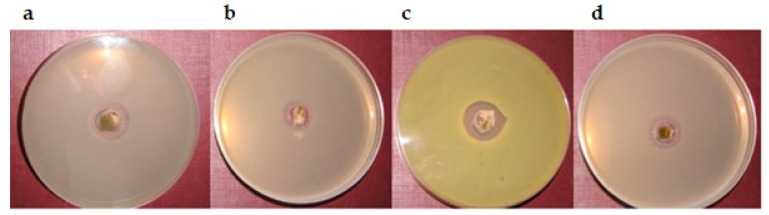
The antimicrobial activity tests of the hydrogels on bacteria: (**a**)—F5 on *P. acnes* (growth inhibition zone 2 mm), (**b**)—F6 on *P. acnes* (growth inhibition zone 2 mm), (**c**)—F6 on *S. aureus* (growth inhibition zone 4 mm), and (**d**)—F6 on *E. coli* (growth inhibition zone 2 mm).

Inflammation and bacteria in the wound cause the formation of exudate, which contains a mixture of cytokines, growth factors, and proteolytic enzymes. Exudate destroys growth factors, body proteins, and the extracellular matrix [91]. Almost all wounds are exposed to non-sterile conditions—hence the high risk of infection. Topical non-antibiotic antimicrobials are preferred to treat wounds, considering the ease of application and rarity of systemic toxicity [92]. The threat of topical antibiotic use is increased resistance bacteria and impaired wound healing [75]. Extract-loaded hydrogels are widely applied as an antibacterial agent in wound healing [93]. The antimicrobial activity of prepared hydrogels was determined for a variety of microorganisms. The hydrogels containing chitosan delivery system with 3% *Calendulae flos* lyophilized extract was found to have a bactericidal effect against *S. aureus*, *P. acnes,* and *E. coli*. Figure 8 shows the diameter of the growth inhibition zone for which the highest hydrogel activity was observed. Antimicrobial activity was not demonstrated for hydrogels based on chitosan delivery system with 10% *Calendulae flos* lyophilized extract, probably due to the high viscosity of the hydrogels. The study confirmed our earlier conclusions that hydrogels containing a chitosan delivery system with 3% *Calendulae flos* lyophilized extract are the most appropriate formulation for the treatment of wounds.

## 4. Conclusions

The hypromellose hydrogel containing a chitosan delivery system with *Calendulae flos* lyophilized extract proved to meet all the criteria for innovative support for the treatment of chronic wounds. The characterization studies of hydrogels were performed by rheological analysis, scanning electron microscopy (SEM), dissolution profiles of active compounds, and antimicrobial activity. Among the hydrogel formulations, the best properties could be attributed to formulation F5 and F6 based on 2% or 3% of HPMC and loaded with 3% of *Calendulae flos* lyophilized extract—chitosan 80/500 in weight ratio 1:1. The synergism action of the active compounds present in the *Calendulae flos* and chitosan was confirmed against hyaluronidase inhibition and antimicrobial properties. The introduction of the *Calendulae flos* lyophilized extract into the chitosan carrier will allow the creation of a hydrogel form pharmaceutical with a controlled release, providing an application algorithm with high compliance response and effectiveness for patients suffering from chronic wounds.

## Figures and Tables

**Table 1:** Composition of HPMC-based hydrogel with chitosan delivery system with *Calendulae flos* lyophilized extract.

Component	F1	F2	F3	F4	F5	F6	F7	F8	F9
*Calendulae flos* lyophilized extract	0.3 g (3%)	0.3 g (3%)	1.0 g (10%)	1.0 g (10%)	-	-	-	-	-
*Calendulae flos* lyophilized extract—chitosan 80/500 1:1	-	-	-	-	0.3 g (3%)	0.3 g (3%)	1.0 g (10%)	1.0 g (10%)	-
*Calendulae flos* lyophilized extract—chitosan 80/1000 1:1	-	-	-	-	-	-	-	-	1.0 g (10%)
HPMC	0.22 g (2%)	0.32 g (3%)	0.2 g (2%)	0.3 g (3%)	0.22 g (2%)	0.32 g (3%)	0.2 g (2%)	0.3 g (3%)	0.22 g (2%)
Water	up 10.0 g	up 10.0 g	up 10.0 g	up 10.0 g	up 10.0 g	up 10.0 g	up 10.0 g	up 10.0 g	up 10.0 g

**Table 2:** Identification phenolic compounds corresponding to the chromatographic peaks in Figure 3 by the UHPLC-DAD and UHPLC-MS/MS methods.

Peak	Rt (min)	UV-VIS (λmax)	[M + H]^+^	*m*/*z* Fragments	Formula	Content mg g^−1^ ± SD	Identification	Mode of Identification
1	9.45	241, 324	-	-	C_16_H_18_O_9_	4.22 ± 0.05	chlorogenic acid	standard
2	14.15	253, 355	771	625, 479, 317	C_34_H_42_O_20_	-	typhaneoside (isorhamnetin 3-O-rhamnosylorutinoside)	literature data
3	17.12	254, 355	625	479, 317	C_28_H_32_O_16_	64.63 ± 0.39	narcissin (isorhamnetin 3-O-rutinoside)	standard

**Table 3:** Apparent permeability values of the chlorogenic acid and the narcissin from *Calendulae flos* lyophilized extract and its chitosan delivery systems.

Substance	*P_app_* ± SD (×10^−6^ cm s^−1^)	
	Chlorogenic Acid	Narcissin
Reference substances	2.42 ± 1.18	2.35 ± 1.46
*Calendulae flos* lyophilized extract	2.90 ± 1.76	1.44 ± 0.68
*Calendulae flos* lyophilized extract—chitosan 80/500 1:1 (*w*/*w*)	2.97 ± 0.01	1.63 ± 0.01
*Calendulae flos* lyophilized extract—chitosan 80/1000 1:1 (*w*/*w*)	3.20 ± 0.41	1.60 ± 0.06

Compounds with *P_app_* < 1 × 10^−6^ cm s^−1^ = low-permeable compound, those with *P_app_* > 1 × 10^−6^ cm s^−1^ = high-permeable compounds [44].

**Table 4:** Values of minimal bactericidal concentration of *Calendulae flos* lyophilized extract and its chitosan delivery systems against selected Gram (+) bacteria, Gram (-) bacteria, and fungi.

Microorganism	*Calendulae flos* Lyophilized Extract	Lyophilized Extract + Chitosan 80/500 1:1	Lyophilized Extract + Chitosan 80/500 1:5	Lyophilized Extract + Chitosan 80/1000 1:1	Lyophilized Extract + Chitosan 80/1000 1:5	Chitosan 80/500	Chitosan 80/1000
	MBC (mg mL^−1^)						
*S. aureus*	8	16	32	16	32	125	250
*S. epidermidis*	8	16	32	16	32	125	250
*E. faecalis*	32	64	125	16	125	125	250
*E. faecium*	32	64	125	32	125	125	250
*S. pyogenes*	64	64	125	32	125	125	250
*E. coli*	64	64	64	32 ↓	64	64	125
*P. aeruginosa*	4	8	64	16	32	125	125
*P. acnes*	16	8 ↓	8 ↓	4 ↓	125	125	125
*Candida albicans*	64	32 ↓	32 ↓	8 ↓	64	250	250

↓—the decrease in MBC value.

**Table 5:** The rheological parameters calculated from steady shear analysis in the controlled rate (CR) mode.

Formulation	Thixotropy	Ostwald’s Model		
		Power-Law Index (*n*)	Consistency (*K*; Pa·s^n^)	Correlation Coefficient
F5	1.9 ± 0.5	0.8491 ± 0.0082	4.87 ± 0.16	0.9998
F6	5.6 ± 2.0	0.7371± 0.0142	23.48 ± 0.57	0.9991
F7	0.9 ± 0.4	0.7940 ± 0.0045	8.24 ± 0.18	0.9995
F8	13.3 ± 4.0	0.6550 ± 0.0037	53.25 ± 0.25	0.998

**Table 6:** The drug flux and the average cumulative amount per area during dissolution studies of HPMC-based hydrogel with chitosan delivery system with *Calendulae flos* lyophilized extract.

Formulation	Drug Flux (*Jss*; µg/cm^2^ h)	Correlation Coefficient (*r*)	Average Cumulative Amount per Area at 7 h (*Q_7h_*; µg/cm^2^)
	Chlorogenic acid		
F1	2.13 ± 0.01	0.9880 ± 0.0570	14.31 ± 0.22
F2	1.70 ± 0.13	0.9950 ± 0.0017	11.21 ± 0.87
F3	6.41 ± 0.36	0.9936 ± 0.0038	43.81 ± 2.71
F4	5.82 ± 0.62	0.9965 ± 0.0019	38.63 ± 3.61
F7	3.04 ± 0.37	0.9923 ± 0.0064	21.23 ± 2.31
F9	2.92 ± 0.28	0.9985 ± 0.0013	19.57 ± 2.02
	Narcissin		
F1	17.54 ± 1.43	0.9945 ± 0.0035	114.29 ± 10.69
F2	13.80 ± 2.09	0.9960 ± 0.0016	97.74 ± 3.38
F3	35.83 ± 2.48	0.9968 ± 0.0008	242.92 ± 18.93
F4	32.84 ± 3.50	0.9964 ± 0.0046	217.59 ± 22.72
F7	26.53 ± 1.57	0.9957 ± 0.0015	179.20 ± 8.69
F9	27.46 ± 3.64	0.9988 ± 0.0013	182.95 ± 24.28

## Supplementary Materials

The following are available online at https://www.mdpi.com/1999-4923/12/7/634/s1, Table S1: Statistical assay of linear plots of the chlorogenic acid and the narcissin, determined by the UHPLC-DAD method. Figure S1: Dissolution studies of *Calendulae flos* lyophilized extract (A) and chitosan (80/500) delivery system with *Calendulae flos* lyophilized extract in weight ratio 1:1 (*w*/*w*) (B) in time 0 min, 30 min, and 180 min by using a standard paddle Agilent 708-DS Dissolution Apparatus with a 150-mL phosphate buffer at pH 5.5 at 32 ± 0.5 °C, Figure S2: Rheological plots obtained for the hydrogel samples in steady shear tests (controlled rate and controlled stress) and oscillatory tests (stress and frequency sweep).
