# Supplementary material for: Hydrogel Delivery System Containing Calendulae flos Lyophilized Extract with Chitosan as a Supporting Strategy for Wound Healing Applications
Source: Pharmaceutics. 2020 Jul 7;12(7):634. doi: 10.3390/pharmaceutics12070634 (PMC7407229; doi:10.3390/pharmaceutics12070634)
Supplement: Supplementary file 1 [file pharmaceutics-12-00634-s001.pdf]

pharmaceutics

Supplementary Materials: Hydrogel Delivery System Containing *Calendulae flos* Lyophilized Extract with Chitosan as a Supporting Strategy for Wound Healing Applications

Justyna Chanaj-Kaczmarek, Magdalena Paczkowska, Tomasz Osmałek, Barbara Kaproń, Tomasz Plech, Daria Szymanowska, Marta Karaźniewicz-Łada, Joanna Kobus-Cisowska and Judyta Cielecka-Piontek

Table S1. Statistical assay of linear plots of the chlorogenic acid and the narcissin, determined by the UHPLC-DAD method.

**Parameter**	**Chlorogenic Acid**		**Narcissin**	
Linearity: y = ax + b				
$a \pm S_a$	0.3963 ± 0.0025		0.1235 ± 0.0050	
$b \pm S_b$	Insignificant		insignificant	
correlation coefficient (r)	0.9999		0.9994	
Limit of detection (LOD): LOD = 3 SD/a (μg mL^{-1})	0.63		10.64	
Limit of quantification (LOQ): LOQ = 10 SD/a (μg mL^{-1})	1.92		32.26	
Range of linearity (μg mL^{-1})	10–100		10–250	
Precision, RSD				
	20 (μg mL^{-1})	1.42	100 (μg mL^{-1})	0.41
	40 (μg mL^{-1})	0.56	150 (μg mL^{-1})	0.67
	60 (μg mL^{-1})	1.31	200 (μg mL^{-1})	1.40
Intra-day, RSD				
	20 (μg mL^{-1})	1.80	100 (μg mL^{-1})	0.96
	40 (μg mL^{-1})	0.75	150 (μg mL^{-1})	1.44
	60 (μg mL^{-1})	1.43	200 (μg mL^{-1})	1.71

where SD is the mean of standard deviations of determinations in the lower range of linearity and *a* is the directional coefficient of the plotted linear function, Sa standard deviation of the slope, Sb standard deviation of the intercept, *t* calculated values of Student's *t* test, $t\alpha$, f = 2.228 critical values of Student's *t* test for degrees of freedom f = 10 and significance level α = 0.05.

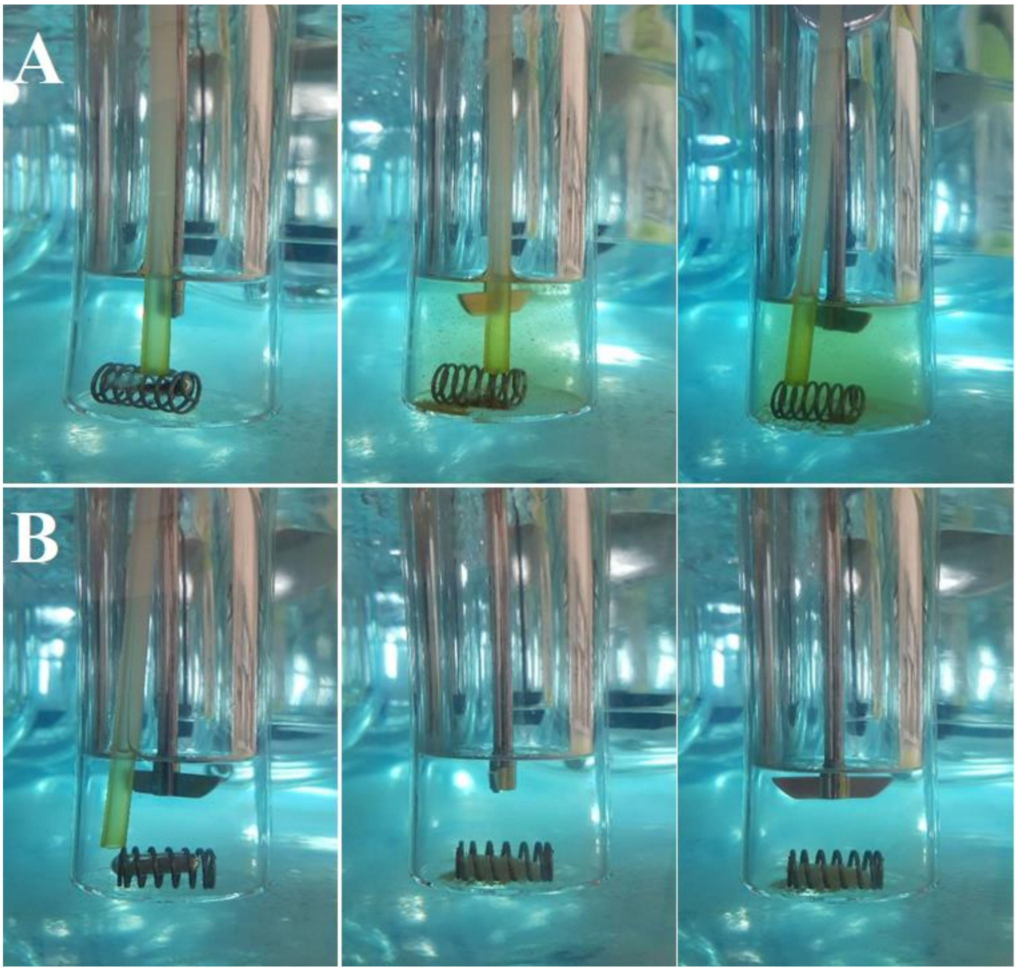

Figure S1. Dissolution studies of *Calendulae flos* lyophilized extract (**A**) and chitosan (80/500) delivery system with *Calendulae flos* lyophilized extract in weight ratio 1:1 (*w*/*w*) (**B**) in time 0 min, 30 min and 180 min by using a standard paddle Agilent 708-DS Dissolution Apparatus with a 150-mL phosphate buffer at pH 5.5 at 32 ± 0.5 °C.

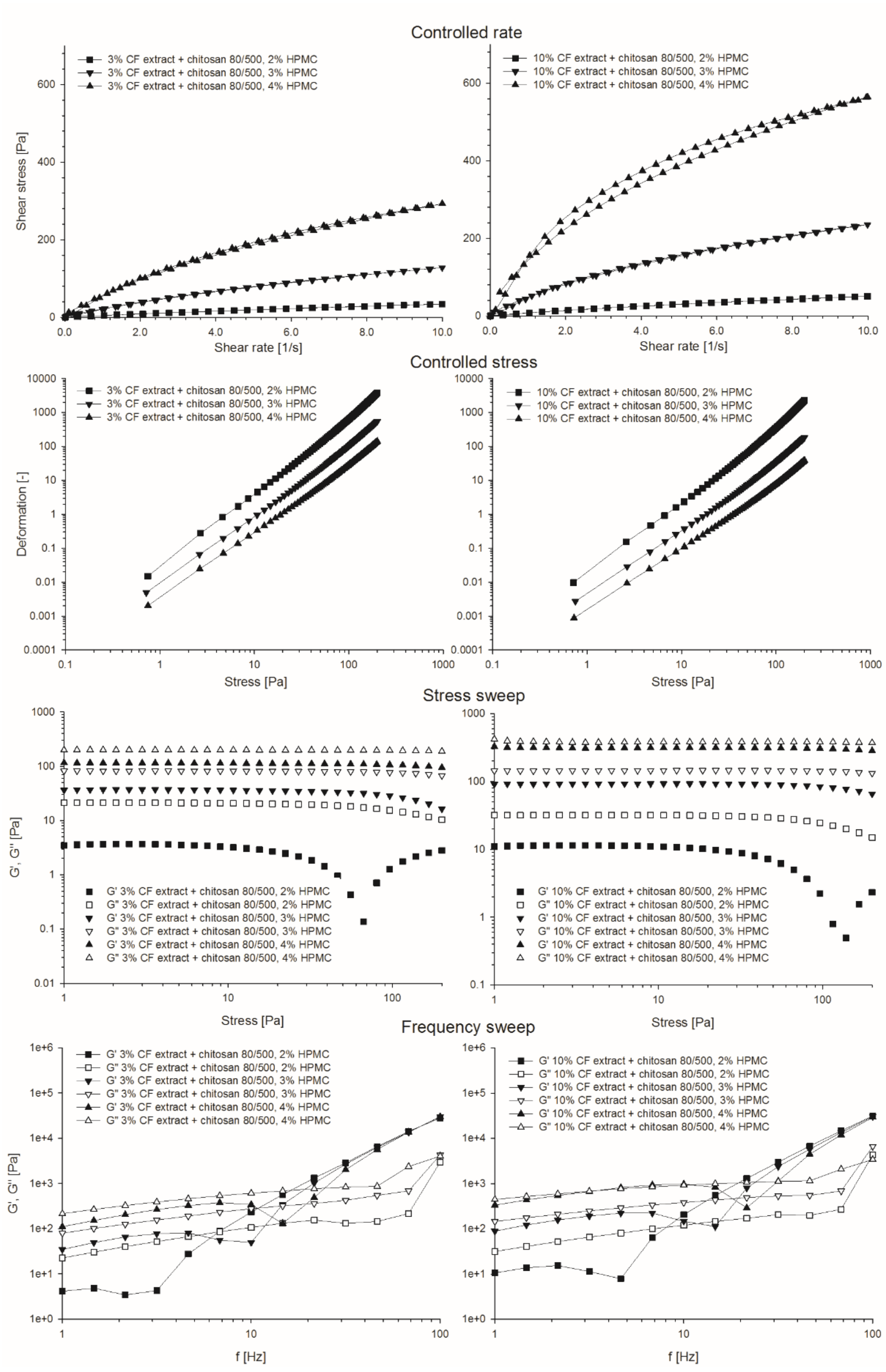

Figure S2. Rheological plots obtained for the hydrogel samples in steady shear tests (controlled rate and controlled stress) and oscillatory tests (stress and frequency sweep).
